# Immune System and Chronic Diseases

**DOI:** 10.1155/2017/4284327

**Published:** 2017-04-30

**Authors:** Margarete Dulce Bagatini, Andréia Machado Cardoso, Alessandra Antunes dos Santos, Fabiano Barbosa Carvalho

**Affiliations:** ^1^Coordenação Acadêmica, Universidade Federal da Fronteira Sul, Chapecó, SC, Brazil; ^2^Department of Molecular Pharmacology, Albert Einstein College of Medicine, New York, NY, USA; ^3^Programa de Pós-graduação em Ciências Biológicas–Bioquímica Toxicológica, Universidade Federal de Santa Maria, Santa Maria, RS, Brazil

Chronic diseases are multifactorial pathological conditions considered as a serious public health problem, accounting for about 60% of deaths worldwide. The immune system has a central role in many processes involving chronic diseases. Immunological research involving cancer, metabolic syndrome, heart diseases, and others has not only enabled the understanding of the mechanisms that underlie these diseases but also suggested new therapies that may impact positively on patients minimizing morbidity and mortality. This special issue aims to present and discuss the advancement of research and innovative therapies involving molecular targets associated with the immune system and chronic diseases as well as the possibility of involving communicable diseases and chronic emerging.

In this special issue, V. Nguyen et al. reviewed the role of interleukin-7 (IL-7) and its receptor in immunosenescence. The review demonstrates that IL-7 is critical to the maintenance of a vigorous health span and longevity. P. Castro-Sánchez et al. investigated gene expression profiles of human phosphotyrosine phosphatases (PTPs) consequent to Th1 polarization and effector function and suggested a regulatory role of autoimmune-related PTPs in controlling T-helper polarization in humans. P. G. Reis et al. investigated possible associations between genetic polymorphisms of IL17A G197A and those of IL17F T7488C with Chagas Disease (CD) and/or the severity of left ventricular systolic dysfunction (LVSD) in patients with chronic Chagas cardiomyopathy (CCC). L.-L. Zhang et al. showed the mechanism whereby poly-L-arginine and lipopolysaccharide can synergistically induce the release of interleukin-6 and interleukin-8 in NCI-H292 cells. In this research, the authors concluded that JNK signaling pathway contributes to the release of IL-6 and IL-8, which is stimulated by the synergistic actions of poly-L-arginine and lipopolysaccharide in NCI-H292 cells.

Idiopathic chronic inflammatory conditions (ICIC) such as allergy, asthma, chronic obstructive pulmonary disease, and various autoimmune conditions are a worldwide health problem. Understanding the pathogenesis of ICIC is essential for an effective therapy and prevention. However, efforts are hindered by the lack of comprehensive understanding of the human immune system function. In this context, S. Balzar describes the concept of stochastic continuous dual resetting (CDR) of the immune repertoire as a basic principle that governs the function of immunity. J. Zheng et al. analyzed the expression of IL-6, TNF-*α*, and MCP-1 in respiratory viral infection in acute exacerbations of a chronic obstructive pulmonary disease.

Inflammation, altered immune cell phenotype, and functions are key features shared by diverse chronic diseases, including cardiovascular, neurodegenerative diseases, diabetes, metabolic syndrome, and cancer. Natural killer (NK) cells are innate lymphoid cells primarily involved in the immune system response to non-self-components but their plasticity is largely influenced by the pathological microenvironment. Altered NK phenotype and function have been reported in several pathological conditions, basically related to impaired or enhanced toxicity. L. Parisi et al. reviewed and discussed the role of NKs in selected, different, and “distant” chronic diseases, cancer, diabetes, periodontitis, and atherosclerosis, placing NK cells as crucial orchestrator of these pathologic conditions.

In this line, to introduce chronic diseases, inflammatory process and new biomarkers, S. Vachatova et al. studied the role of selected inflammatory and anti-inflammatory serum markers of psoriatic patients in the pathogenesis of metabolic syndrome (MetS) and psoriasis. The study was based on the comparison between the group of psoriatic patients and the control group. S. Akarsu et al. compared the frequencies of MetS and its components in discoid lupus erythematosus (DLE) with the non-DLE control group. Additionally, the authors determined the differences of sociodemographic and clinical data of the DLE patients with MetS compared to that of the patients without MetS. This was a cross-sectional, case-control study. C. D. P. Júnior et al. studied the influence of the expression of inflammatory markers on the kidney after fetal programming in an experimental model of renal failure. A study carried out by O. Khalifa et al. observed the influence of the geographic origin in the TMEM187-IRAK1 polymorphisms associated with rheumatoid arthritis susceptibility in Tunisian and French female populations. Y. Araki and T. Mimura reviewed the epigenetic mechanisms such as histone modifications, DNA methylation, and microRNAs in the development of rheumatoid arthritis (RA). The review shows that aberrant epigenetic changes contribute to the development of RA and affect disease susceptibility and severity. A cross-sectional study conducted by J.-K. Kim et al. demonstrated that the neutrophil extracellular trap (NET) formation might be changed in end-stage renal disease (ESRD) patients, explaining their higher incidence of coronary artery diseases (CAD). In addition, the uremia-associated-increased NET formation may be a sign of increased burden of atherosclerosis.

J. Wu et al. reported 48 chronic granulomatous disease (CGD) patients in a single-center study from Mainland China. They identified 39 gene mutations in these patients, including 36 mutations in CYBB gene, 1 mutation in CYBA gene, 1 mutation in NCF1 gene, and 1 mutation in NCF2 gene. They found that the compulsory Bacillus Calmette-Guerin (BCG) vaccination for all infants after birth contributes to the early onsets in CGD patients in Mainland China. The study carried out by S. J. Lee et al. showed that the serum levels of 25-hydroxyvitamin D3 were significantly lower in patients with primary Sjogren's syndrome (SS) compared with those in age- and sex-matched sicca controls. Furthermore, Sjogren's syndrome disease activity index (ESSDAI) was negatively associated with serum levels of 25(OH)-D3 and positively associated with B-cell activation of the tumor necrosis factor family (BAFF). J. Zhao et al. demonstrated the differences in antibody responses to mycobacterium tuberculosis antigens in Japanese tuberculosis patients infected with the Beijing/non-Beijing genotype. M. R. Al-Anazi et al. showed the association of Toll-like receptor 3 single nucleotide polymorphisms and hepatitis C virus infection. Y. Han et al. demonstrated that there is an impairment of ex vivo phagocytic function of macrophages from myelodysplastic syndrome (MDS) patients. The authors also showed levels of reorganization receptors CD206 and SIRP*α* were lower accompanied by an increase in the iNOS levels secreted by macrophages in MDS. These findings show that monocyte-derived macrophages are impaired in MDS. J. E. Kim et al. reviewed the medical records of 30 patients with anetoderma. The results suggest that protruding type anetoderma may represent a more advanced stage, and the metalloproteinase- (MMP-) 2 and MMP-9 could be responsible for elastic fiber degradation in anetoderma.

The review article by A. Mietelska-Porowska and U. Wojda highlighted the alterations concerning T lymphocytes and immune mediators such as cytokines and chemokines in Alzheimer's disease brain, cerebrospinal fluid, and blood and the mechanisms by which peripheral T cells cross the blood brain barrier and the blood-CSF barrier. L. Frick and C. Pittenger showed in original review evidences that immune dysregulation contributes to the pathophysiology of obsessive-compulsive disorder, Tourette syndrome, and pediatric autoimmune neuropsychiatric disorders associated with streptococcal infections. The authors explore new hypotheses as to the potential contributions of microglial abnormalities to pathophysiology, beyond neuroinflammation, failures in neuroprotection, lack of support for neuronal survival, and abnormalities in synaptic pruning.

C. Auriti et al. discussed the genetic and biologic characteristics of mannose-binding lectin, a member of the collecting family, and its role in the susceptibility to infections and to ischemia-reperfusion-related tissue injuries during the perinatal period and the possible future therapeutic applications. P. M. Becher et al. compared the acute and chronic phases of viral myocarditis to identify the immediate effects of cardiac inflammation as well as the long-term effects after resolved inflammation on cardiac fibrosis and consequently on cardiac function.

The topic of immunological research and cancer was extensively discussed. P. H. Pandya et al. provided the framework for a novel subspecialty known as immunooncology This review highlights the fundamentals of incorporating precision medicine to discover new immune biomarkers and predictive signatures. A. M. Mehta et al. described in a review that multiple genes and loci are associated with variation in risk of cervical cancer. Rather than one single gene being responsible for cervical carcinogenesis, the authors postulate that variations in the different immune response genes lead to subtle differences in the effectiveness of the antiviral and antitumor immune responses, ultimately leading to differences in risk of developing cervical cancer and progressive disease after HPV infection. L. M. Barajas-Castañeda et al. analyzed the overexpression of MMP-3 and uPA with diminished PAI-1 related to metastasis in ductal breast cancer patients attending a public hospital in Mexico City. D.-W. Yeh et al. showed the role of Toll-like receptor signaling in the interplay between inflammation and stemness in cancer cells.

Innovative therapies were also a highlighted topic in this special issue. The study by S. F. G. Zorzella-Pezavento et al. demonstrated that previous vaccination with pVAXhsp65 was able to reduce experimental autoimmune encephalomyelitis (EAE) clinical manifestations in C57BL/6 mice and triggered higher IL-10 production at the central nervous system. The review article by H. Mitoma et al. indicates that epitope-specific GAD65Ab-induced impairment of GABA release is involved in the pathogenesis of GAD65Ab-positive cerebellar ataxias (CAs) and supports the early detection of GAD65Ab-associated CA to initiate immunotherapy before irreversible neuronal death in the cerebellum. I. Rohm et al. showed that treatment with ivabradine in patients with chronic heart failure resulted in an improvement of heart failure symptoms and ejection fraction as well as a normalization of inflammatory mediators. A study conducted by L. Mattson et al. suggested the potential involvement of type I interferon signalling in immunotherapy in seasonal allergic rhinitis. B. Ye et al. reviewed the genetically modified T cell-based adoptive immunotherapy in hematological malignancies. A. Navinés-Ferrer et al. focus on IgE-related chronic diseases, such as allergic asthma and chronic urticaria (CU), and on the role of the anti-IgE monoclonal antibody, omalizumab, in their treatment. Y. Zhou et al. demonstrated that dendritic cells modified by tofacitinib exhibited a typical tolerogenic phenotype, and Tofa-DCs pulsed with myelin oligodendrocyte glycoprotein (MOG)_35–55_ could effectively dampen the severity and progression of experimental autoimmune encephalomyelitis (EAE). This suppression of EAE was associated with a remarkable decrease of Th1/Th17 cells and an increase in regulatory T cells (Tregs).

I.-S. Lee et al. demonstrated the anti-inflammatory effects of ginsenoside Rg3 via inhibiting NF-kappa B activation in IL-1*β*-induced A549 cells and human asthmatic lung tissue. A study conducted by X. Meng et al. verified the hyperlipidemia caused by overuse of glucocorticoid after liver transplantation and the immune adjustment strategy. J. Wang et al. provide a detailed overview about an important consideration that long-term administration of DT induces a humoral immune response with an appropriate production of anti-DT antibodies that can inactivate DT and thus abrogate its effect in the DEREG mouse. Additionally, the authors showed that anti-DT mouse serum partially neutralized DT-induced Foxp3 inhibition. H. Dong et al. present a brief summary of the defensins in immunity with specific reference to human and animal tuberculosis and explore their potential as a novel approach to therapy or prophylaxis.

*Margarete Dulce Bagatini* *Margarete Dulce Bagatini* *Andréia Machado Cardoso* *Andréia Machado Cardoso* *Alessandra Antunes dos Santos* *Alessandra Antunes dos Santos* *Fabiano Barbosa Carvalho* *Fabiano Barbosa Carvalho*

